# A Deep Learning Approach for Rapid and Generalizable Denoising of Photon-Counting Micro-CT Images

**DOI:** 10.3390/tomography9040102

**Published:** 2023-07-02

**Authors:** Rohan Nadkarni, Darin P. Clark, Alex J. Allphin, Cristian T. Badea

**Affiliations:** Quantitative Imaging and Analysis Lab, Department of Radiology, Duke University Medical Center, Durham, NC 27710, USA; rohan.nadkarni@duke.edu (R.N.); darin.clark@duke.edu (D.P.C.); alex.allphin@duke.edu (A.J.A.)

**Keywords:** denoising, deep learning, preclinical, micro-CT, photon-counting CT, contrast agents

## Abstract

Photon-counting CT (PCCT) is powerful for spectral imaging and material decomposition but produces noisy weighted filtered backprojection (wFBP) reconstructions. Although iterative reconstruction effectively denoises these images, it requires extensive computation time. To overcome this limitation, we propose a deep learning (DL) model, UnetU, which quickly estimates iterative reconstruction from wFBP. Utilizing a 2D U-net convolutional neural network (CNN) with a custom loss function and transformation of wFBP, UnetU promotes accurate material decomposition across various photon-counting detector (PCD) energy threshold settings. UnetU outperformed multi-energy non-local means (ME NLM) and a conventional denoising CNN called UnetwFBP in terms of root mean square error (RMSE) in test set reconstructions and their respective matrix inversion material decompositions. Qualitative results in reconstruction and material decomposition domains revealed that UnetU is the best approximation of iterative reconstruction. In reconstructions with varying undersampling factors from a high dose ex vivo scan, UnetU consistently gave higher structural similarity (SSIM) and peak signal-to-noise ratio (PSNR) to the fully sampled iterative reconstruction than ME NLM and UnetwFBP. This research demonstrates UnetU’s potential as a fast (i.e., 15 times faster than iterative reconstruction) and generalizable approach for PCCT denoising, holding promise for advancing preclinical PCCT research.

## 1. Introduction

Photon-counting detectors (PCDs) offer the potential to improve performance of CT imaging at both clinical and preclinical levels and to extend the use of CT to novel applications [1]. PCDs acquire images at two or more energies simultaneously by detecting individual x-ray photons and sorting them into bins based on energy thresholds, making photon-counting CT (PCCT) a promising tool for spectral imaging tasks that require material decomposition. Moreover, PCD energy thresholds can be adjusted near the K-edges of various contrast agents, enabling their spectral separation in novel imaging procedures [2,3].

However, PCCT dose constraints and photon binning result in high noise levels, particularly in high-energy bins, reducing image quality and the accuracy of material decomposition. Multi-energy CT can be denoised using various post-reconstruction algorithms such as non-local means (NLM) filtering [4,5]. Alternatively, lower noise can be achieved via iterative reconstruction [6,7,8] or deep learning (DL)-based methods [9]. In our own work, iterative reconstruction algorithms such as those using rank-sparse kernel regression (RSKR) [10] are effective at producing low-bias denoised PCCT reconstructions that result in highly accurate material decomposition. However, such iterative approaches are computationally expensive.

To address this limitation, DL models can be used to approximate iterative reconstruction, providing robust denoising with reduced computation time. Commonly, DL methods use convolutional neural networks (CNNs) to learn the mapping between noisy and clean images directly from data, eliminating the need for explicit knowledge of the underlying statistical model. Previous studies have demonstrated that DL-based denoising can achieve comparable performance to iterative reconstruction, with significantly reduced computational time [11]. Various DL architectures have been used for denoising CT images, including U-nets [12], residual encoder–decoder (RED) CNN [13], generative adversarial networks (GANs) [14], and vision transformers [15]. U-nets are a popular choice for CT image denoising [16,17,18,19,20] because they offer several advantages. These include fast computation at test time due to a relatively small number of parameters and simple architecture, an encoder–decoder structure that allows for efficient feature extraction and information propagation, and skip connections between encoder and decoder that allow the network to retain and use both high- and low-level features during the denoising process [21]. Nevertheless, challenges remain in DL-based denoising, such as the risk of overfitting to training data, leading to limited generalization performance. Generalizability is crucial when denoising PCCT images, given that PCD energy thresholds change based on the contrast agents used, affecting the noise level in each energy channel. Furthermore, there is a need for robust evaluations that can accurately measure the performance of DL-based denoising algorithms.

In this study, we introduce a supervised learning approach to develop a denoising CNN that adapts to various PCD threshold settings. Our goal is to perform fast and reliable denoising of weighted filtered backprojection (wFBP) reconstructions at the level of iterative reconstruction in scans acquired on our in-house developed preclinical PCCT imaging system [22] with four energy thresholds. We trained UnetU, which is a U-net CNN with a unique preprocessing method and loss function intended to promote generalizability and accurate material decomposition. Then, we compared UnetU to a traditional U-net denoising approach called UnetwFBP as well as a non-data-driven filtering method called multi-energy non-local means (ME NLM). Instead of proposing a novel CNN architecture, our study contributes to a growing body of DL methods for denoising micro-CT data by introducing a unique input preprocessing approach and loss function that enable a well-known CNN such as the U-net to produce accurate reconstructions and material separation at various PCD energy threshold settings.

## 2. Materials and Methods

### 2.1. Image Acquisition

For this study, we acquired all PCD image data using a dual-source micro-CT imaging system consisting of an energy-integrating detector (EID) and a PCD (Dectris Santis 1604, 150 μm pixel size, 4 energy thresholds) [22]. We used a G-297 x-ray tube (Varian Medical Systems, Palo Alto, CA, USA) with a 0.3 mm focal spot size and a 0.1 mm Cu filter and utilized an Epsilon high-frequency x-ray generator (EMD Technologies, Saint-Eustache, QC, Canada). We used only PCD-based acquisitions for the scans in this study.

The PCCT scans used in this work are summarized in Table 1. Our CNN training and validation sets consisted of data from 10 different helical scans of mice (9 ex vivo, 1 in vivo) that provided 4 distinct PCD threshold settings and 5 different contrast agents: Iodine (I), Gadolinium (Gd), Tantalum (Ta), Bismuth (Bi), and Gold (Au). The testing set included 5 different scans: 2 scans of phantoms containing vials of known material concentrations, 1 ex vivo mouse, and 1 in vivo mouse to provide sets at each of the threshold settings included in training, and an additional in vivo scan of a mouse with thresholds 28, 34, 39, 45 keV, and contrast agents I and Barium (Ba) to determine how our CNNs generalized to threshold settings not included in training. PCD energy thresholds were chosen to be slightly above the K-edges of contrast agents that were used as basis materials in the decomposition of the PCCT reconstruction. Each scan was acquired using a helical trajectory with 1070 degrees rotation. The number of projection angles for the helical scans varied between 900 and 1500, and vertical translation varied between 1.25 and 2.5 cm.

### 2.2. Image Reconstruction

All PCD micro-CT scan data underwent reconstruction using the weighted filtered backprojection (wFBP) algorithm [23] with an isotropic voxel size of 125 microns. Additionally, the PCCT data were jointly reconstructed using a multi-channel iterative algorithm to create training labels and for comparison with ME NLM, UnetwFBP, and UnetU denoising in the test set. Specifically, we applied the split Bregman method with the add-residual-back strategy [24] and rank-sparse kernel regression (RSKR) regularization [10]. Reconstruction iteratively solved the following equation:

(1) $$X=\underset{X}{arg\;\min}\frac{1}{2}\;\underset{e}{\sum }{\left|\left|\;RX\left(e\right)-Y\left(e\right)\;\right|\right|}_{2}^{2}+\lambda {\left|\left|\;X\;\right|\right|}_{BTV}.$$

The reconstructed data (*X*) at each energy (*e*) minimizes the reprojection error (*R* representing the system projection matrix) relative to log-transformed projection data (*Y*). To reduce noise in the reconstructed results, this data fidelity term is minimized subject to the bilateral total variation (*BTV*). *BTV* measured within and between energies is reduced via RSKR.

### 2.3. Material Decomposition

The method of material decomposition was applied to the wFBP and iterative RSKR reconstructions, as well as ME NLM, UnetwFBP, and UnetU denoised reconstructions. We performed image-based material decomposition using the method of Alvarez and Macovski [25] extended to include K-edge contrast materials. Thus, we performed a post-reconstruction spectral decomposition with H_2_O, Calcium (Ca), and *N* K-edge materials as basis functions, $M_{i}$:

(2) $$X\left(e\right)=C_{H2O}M_{H2O}\left(e\right)+C_{Ca}M_{Ca}\left(e\right)+\overset{N}{\underset{i=1}{\sum }}C_{i}M_{i}\left(e\right).$$

In this formulation, *M* is a matrix of material sensitivities at each energy. We computed these values by fitting the slope of attenuation measurements taken in vials of known material concentrations. *C* represents density in g/mL for water and concentration in mg/mL for all other materials. Finally, *X* is the attenuation coefficient of the voxel under consideration at energy *e*. Material decomposition was performed by matrix inversion, solving the following linear system at each voxel:

(3) $$C=XM^{-1}\;$$

Orthogonal subspace projection was used to prevent negative concentrations [10]. Post decomposition, the material maps were assigned to colors and combined and visualized in ImageJ.

### 2.4. Network Training and Testing

**Figure 1:**
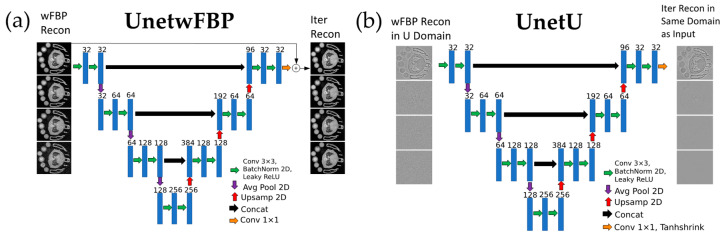
Diagram of CNN architecture and training procedure for each denoising U-net. (**a**) UnetwFBP, a denoising U-net approach with conventional choice of network input and labels. (**b**) UnetU, a denoising U-net approach augmented by our novel input preprocessing method.

In Section 2.1, we allocated ten sets of reconstructions for training and validation, and reserved five sets for testing. We implemented two 2D U-net [26] CNNs (with four levels and two convolutions per level) using the PyTorch library [27] and compared their performance. Figure 1 illustrates the implementation of these U-nets.

Our first U-net, named UnetwFBP, represents a conventional U-net denoising approach that serves as a baseline for comparison. UnetwFBP uses wFBP reconstructions as input and iterative RSKR reconstructions as its training labels. Given the non-zero-centered nature of the input data, we included a residual connection between the input and output in UnetwFBP to address intensity bias. We trained UnetwFBP using a mean squared error (MSE) loss function.

The second U-net, named UnetU, is a novel approach that improves the ability of a standard U-net CNN to perform generalizable PCCT denoising through our unique preprocessing of the wFBP input. First, we divide each energy channel of the wFBP image by its noise variance measured as the Median Absolute Deviation (MAD) of image intensity gradients [28] in the central axial slice. Then, we compute the singular value decomposition (SVD) along the energy dimension. The noise variance weighted SVD helps to compensate for the noise level of each channel to promote generalizability, while creating a representation with less redundancy between channels than the wFBP reconstruction. Next, each left singular vector image from the SVD is high-pass-filtered by subtracting a copy of itself that has been blurred with a 3D Gaussian filter of kernel size 37 × 37 × 37 and full width at half maximum (FWHM) of 10 voxels. This filtering helps accelerate training convergence by making the network input zero-centered to match the non-linear activation functions and initialization of batch normalization in the U-net. Furthermore, it promotes generalization because high-frequency information in PCCT images is more consistent across different energy threshold settings than low-frequency information. Finally, each singular vector is divided by its absolute 99.95 percentile intensity value to create the images (U) that are used as the input to UnetU. This division brings the extremely small variance of each high-pass-filtered singular vector much closer to the variance of 1 that is assumed during initialization of U-net parameters. We used the following output activation function for UnetU:

(4) Tanhshrink(x)=x−tanh(x)

to help reduce the bias of network predictions for large negative and positive values in the U images. Although our transformation is applied globally for each 4D image volume, our loss function exploited the fact that it can be inverted for individual axial slices at all 4 energies. We trained UnetU using the following four-term loss function, with labels derived from iterative RSKR reconstruction:

(5) $$Loss=\frac{1}{N}∥X-X_{label}∥{}_{F}^{2}+\lambda_{1}\frac{1}{N}∥U-U_{label}∥{}_{F}^{2}+\lambda_{2}\frac{1}{N}∥XM^{-1}-X_{label}M^{-1}∥{}_{F}^{2}+\frac{1}{N}∥X_{blur}-X_{blur,\;label}∥{}_{F}^{2}$$

where *N* is the number of voxels, *X* and *X_label_* are the prediction and label in the reconstruction domain, *U* and *U_label_* are the prediction and label in the normalized, high-pass-filtered left singular vector domain of the wFBP, *M* is the sensitivity matrix used for material decomposition, and *X_blur_* and *X_blur,label_* are the prediction and label in the reconstruction domain after blurring axial slices with a 2D Gaussian filter of kernel size 19 × 19 and FWHM 5. Although the first term in the loss function already penalizes error in the reconstruction domain, the fourth term further increases penalization of bias in attenuation values. To ensure that the material decomposition loss term penalizes each material map at a similar intensity scale, the sensitivity matrices (*M*) used in the loss function were rescaled via multiplication of each row by the corresponding calibration vial concentration. To avoid problems such as excessively long training time per epoch, discrepancies between the magnitudes of cost function terms, and vanishing gradients associated with thresholding, the decompositions in the loss function did not incorporate a non-negativity constraint. We used $\lambda_{1}=15$ and $\lambda_{2}=0.2$ to give the first 3 loss terms similar magnitude. In addition to training UnetU with the custom loss function in Equation (5), we also trained UnetU with only the first term of Equation (5) (MSE loss) to allow us to assess how the custom loss function affects test set performance.

The UnetwFBP and UnetU models both take 2D axial image slices of size 400 × 400 voxels with 4 channels as the input. Both networks have 4 input channels and 4 output channels to match the number of PCD energy thresholds. We held out one set of matching wFBP and iterative reconstruction volumes as a validation set, with the remaining nine sets used for training. Then, we randomly shuffled the order of the slices in both the training and validation sets. Data augmentation for both UnetwFBP and UnetU consisted of randomly flipping input image slices horizontally and vertically with 50% probability for each flip. For UnetU, we randomly multiplied both the left and right singular vectors by –1 with 50% probability as an additional form of data augmentation. All U-nets were trained to run for a maximum of 2000 epochs with a batch size of 8, Adam optimizer [29], an initial learning rate of 2 × 10^−4^, a learning rate scheduler, and early stopping. The learning rate scheduler cuts the learning rate in half every time the validation loss fails to decrease for 10 consecutive epochs, and early stopping terminates training when the validation loss fails to decrease for 20 consecutive epochs. UnetwFBP, UnetU with MSE loss, and UnetU with custom loss were trained on a computer running Linux with four NVIDIA RTX 6000 Ada GPUs. After training was completed, all three models were used to predict estimates of iterative RSKR reconstruction for the test sets described in Section 2.1.

### 2.5. Performance Evaluation

We conducted a thorough evaluation of the performance of the UnetwFBP and UnetU approaches, considering computation time, quantitative metrics, qualitative results, and performance at varying radiation doses. For all analyses, UnetwFBP and UnetU were compared both to each other and to the ME NLM algorithm. As suggested in the original paper [5], our ME NLM implementation uses a search window W_Ω_ = 11^3^ voxels, patch size W_p_ = 3^3^ voxels, filtering strength h = 1.2, and noise standard deviation values at each energy derived from homogeneous region of interest (ROI) measurements (from a water vial). Each time we call the ME NLM function, we pass in 31 adjacent axial slices of the wFBP image and take the filtered version of the 16th axial slice as the output.

To assess computational burden, we measured the time required for iterative RSKR reconstruction, ME NLM denoising, UnetwFBP prediction, and UnetU prediction on one of the test sets.

Our quantitative metrics included average bias in Hounsfield Units (HU) relative to wFBP for each denoising method (iterative RSKR, ME NLM, UnetwFBP, UnetU with MSE loss, UnetU with custom loss) and average noise level in HU for wFBP and each denoising method. For each of the 5 test sets, we chose an axial slice with a water vial, defined an ROI within the vial, and measured the mean and standard deviation in this ROI for wFBP and each denoising method at all four energy thresholds. To determine average bias for each denoising method at each energy threshold, we computed the absolute difference between mean value in the water vial ROI from denoised reconstruction and from wFBP in each test set and took the mean of the 5 absolute difference values. The average noise level for wFBP and for each denoising method at each energy threshold was calculated by taking the mean of the 5 standard deviation values from water vial ROIs in the test sets. In addition to computing average bias and noise metrics in the reconstructions, we compared the average root mean square error (RMSE) in HU relative to iterative RSKR reconstruction across the 5 test sets for wFBP reconstruction, ME NLM, UnetwFBP, UnetU with MSE loss and UnetU with custom loss.

For each test set, we used the sensitivity matrix derived from calibration vial measurements in the iterative RSKR reconstruction to convert reconstructions from wFBP and each denoising method into material decompositions via sensitivity matrix inversion with the approach described in Section 2.3. Then, we computed the average test set RMSE of wFBP, ME NLM, UnetwFBP, UnetU with MSE loss, and UnetU with custom loss in the material maps relative to the decompositions from iterative reconstruction. Since quantitative comparisons of bias, noise level, and RMSE in reconstructions and material maps are sufficient to demonstrate the effect of loss function selection on UnetU, the remaining analyses only include UnetU with the custom loss function.

For qualitative comparison, we show reconstructions and corresponding matrix inversion material decompositions from iterative RSKR, wFBP, ME NLM, UnetwFBP, and UnetU on a test set scan of a phantom containing vials of water and known concentrations of the K-edge contrast materials Gd, Ta, and Bi. This set was chosen because of the very low photon counts and resulting high noise level associated with the high energy thresholds corresponding to the K-edges of its contrast agents (e.g., K-edge at ~90 keV for Bi). In addition, we show reconstructions and the corresponding matrix inversion decompositions on a test set in vivo scan of a mouse with tumors that was injected both with I and Ba-based contrast agents to demonstrate how our U-net denoising generalizes to threshold settings not included in training. Like our RMSE analysis, these qualitative comparisons treat iterative RSKR reconstruction and its matrix inversion decomposition as the ground truth. Therefore, we also display absolute subtraction maps from the iterative reconstruction for ME NLM, UnetwFBP, and UnetU in order to make their artifacts more visible. The conclusions we draw from these qualitative analyses are based on the assessment of small animal imagers on our team (CTB, DPC) who have more than a decade of experience.

To evaluate each denoising method at different dose levels, we acquired a helical scan with I and Gd K-edge threshold settings of an ex vivo mouse with 2700 projections, which is about 3 times the dose level of a typical preclinical in vivo helical scan (using 900 projections with dose 36 mGy). We performed wFBP reconstruction of the full set of 2700 projections and projection data generated by undersampling factors of 2, 3, 4, 5, and 6 to simulate a wide range of radiation dose levels. For each of the 6 wFBP reconstructions, we used iterative RSKR reconstruction, ME NLM, UnetwFBP, and UnetU to denoise the images. We show wFBP and denoised images from the highest energy threshold (60 keV) for the fully sampled, 3x undersampled, and 6x undersampled cases in an ROI from a coronal slice. We also plot structural similarity (SSIM) and peak signal-to-noise ratio (PSNR) values in this slice for each denoising method at each undersampling factor relative to the iterative RSKR reconstruction of the complete set of 2700 projections.

## 3. Results

### 3.1. Loss Curves

**Figure 2:**
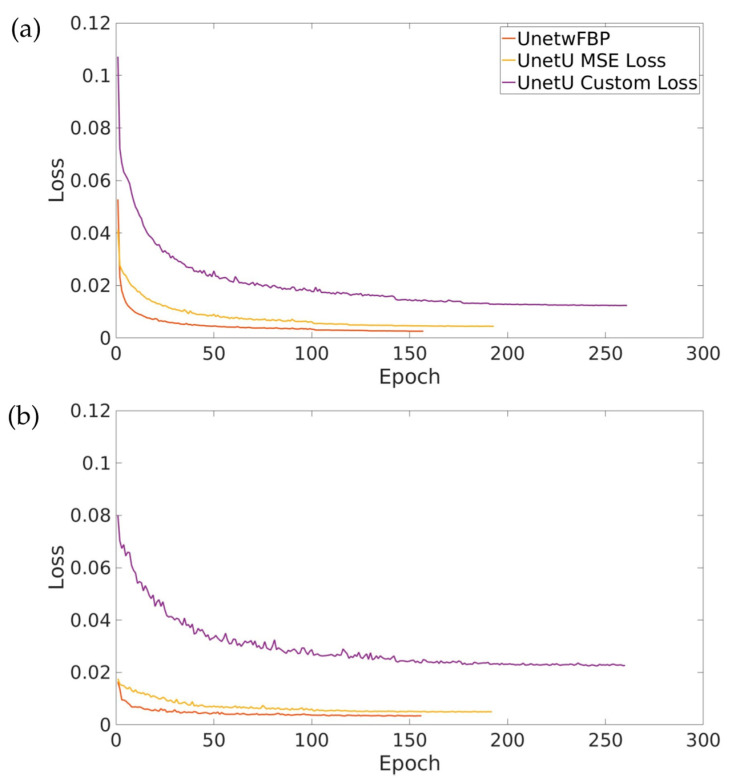
Training (**a**) and validation (**b**) loss curves for all three denoising U-nets.

Figure 2 shows the training and validation loss curves for UnetwFBP, UnetU with MSE loss, and UnetU with custom loss. All three networks completed training within 300 epochs due to the early stopping implementation. Training took 8 h and 42 min for UnetwFBP, 11 h and 9 min for UnetU with MSE loss, and 16 h and 56 min for UnetU with custom loss.

### 3.2. Computation Time

On the same computer used to train the U-nets, iterative RSKR reconstruction took 915 s on a test set with dimensions of 400 × 400 × 400 voxels and 4 energy thresholds, while ME NLM took 854 s, UnetwFBP took 36 s, and UnetU took 61 s to denoise the same test set. Please note that our ME NLM implementation is not optimized for speed, and it is likely that greater reduction in computation time can be achieved with ME NLM.

### 3.3. Quantitative Analyses

**Figure 3:**
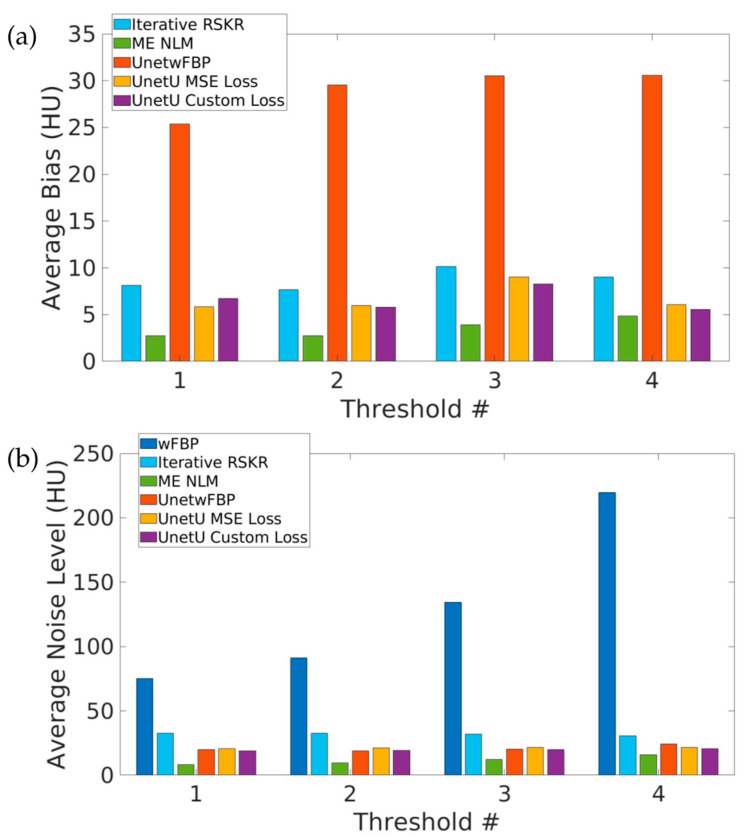
Average bias (**a**) and noise level (**b**) in water vial measurements from test set images.

Figure 3 shows the average bias relative to wFBP as well as the average noise level of wFBP and the denoising methods at each of the four PCD thresholds in water vial measurements. All denoising approaches except UnetwFBP have an average bias of less than 11 HU at all four energy thresholds. Iterative RSKR reconstruction, ME NLM, and all three U-nets give an average noise level of less than 35 HU at all four thresholds, while the average noise level of wFBP reconstruction is above 70 HU at all four thresholds. ME NLM has the lowest bias as well as the lowest noise level. Compared to UnetU with MSE loss, UnetU with custom loss shows lower bias for three out of four PCD thresholds and lower noise level for all four thresholds.

**Figure 4:**
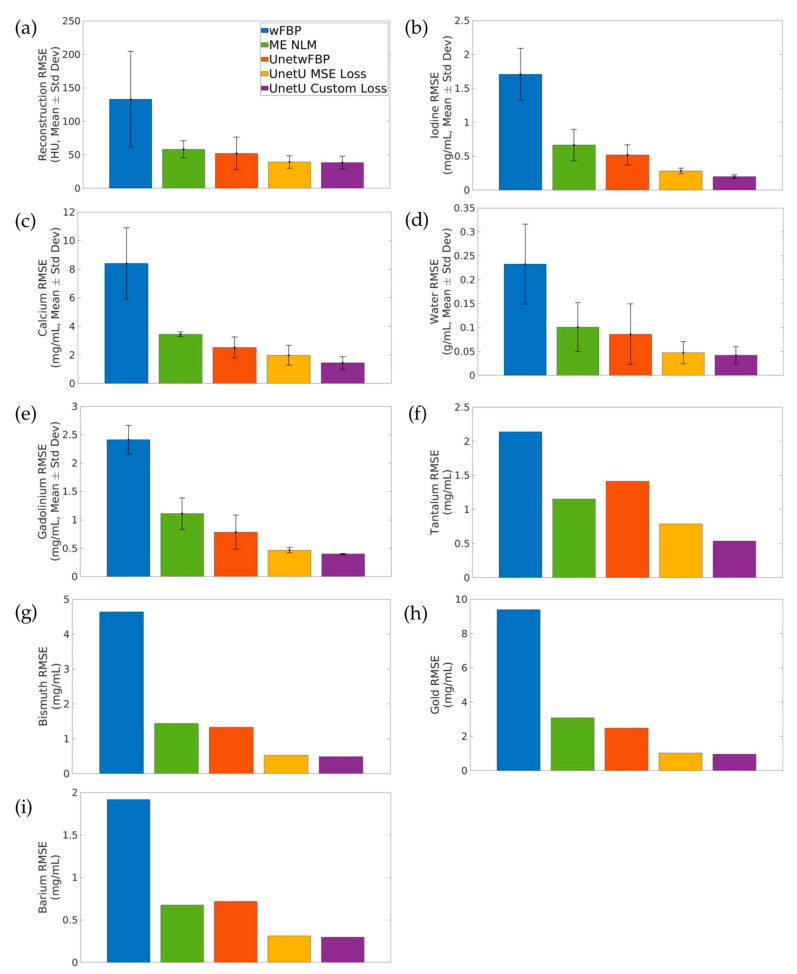
Average root mean square error (RMSE) of wFBP and each denoising method relative to iterative RSKR reconstruction and its matrix inversion decompositions. (**a**) Reconstruction; (**b**) Iodine; (**c**) Calcium; (**d**) Water; (**e**) Gadolinium; (**f**) Tantalum; (**g**) Bismuth; (**h**) Gold; (**i**) Barium. Error bars indicate standard deviation. Error bars are not included in the plots for Tantalum, Bismuth, Gold, or Barium because these materials are only present in the decomposition of one test set.

Figure 4 shows the average test set RMSE of wFBP, ME NLM, and the denoising U-nets relative to iterative RSKR reconstruction and its matrix inversion decompositions. ME NLM and all three U-nets provide dramatically lower error than the wFBP reconstruction and its decomposition. UnetU with custom loss is most accurate on average in the reconstruction (38.631 ± 9.270 HU) and consistently provides the lowest RMSE in the material maps (0.200 ± 0.026 mg/mL for Iodine, 1.445 ± 0.436 mg/mL for Calcium, 0.042 ± 0.018 g/mL for Water, 0.399 ± 0.010 mg/mL for Gadolinium, 0.537 mg/mL for Tantalum, 0.491 mg/mL for Bismuth, 0.959 mg/mL for Gold, 0.297 mg/mL for Barium).

### 3.4. Vials Phantom

**Figure 5:**
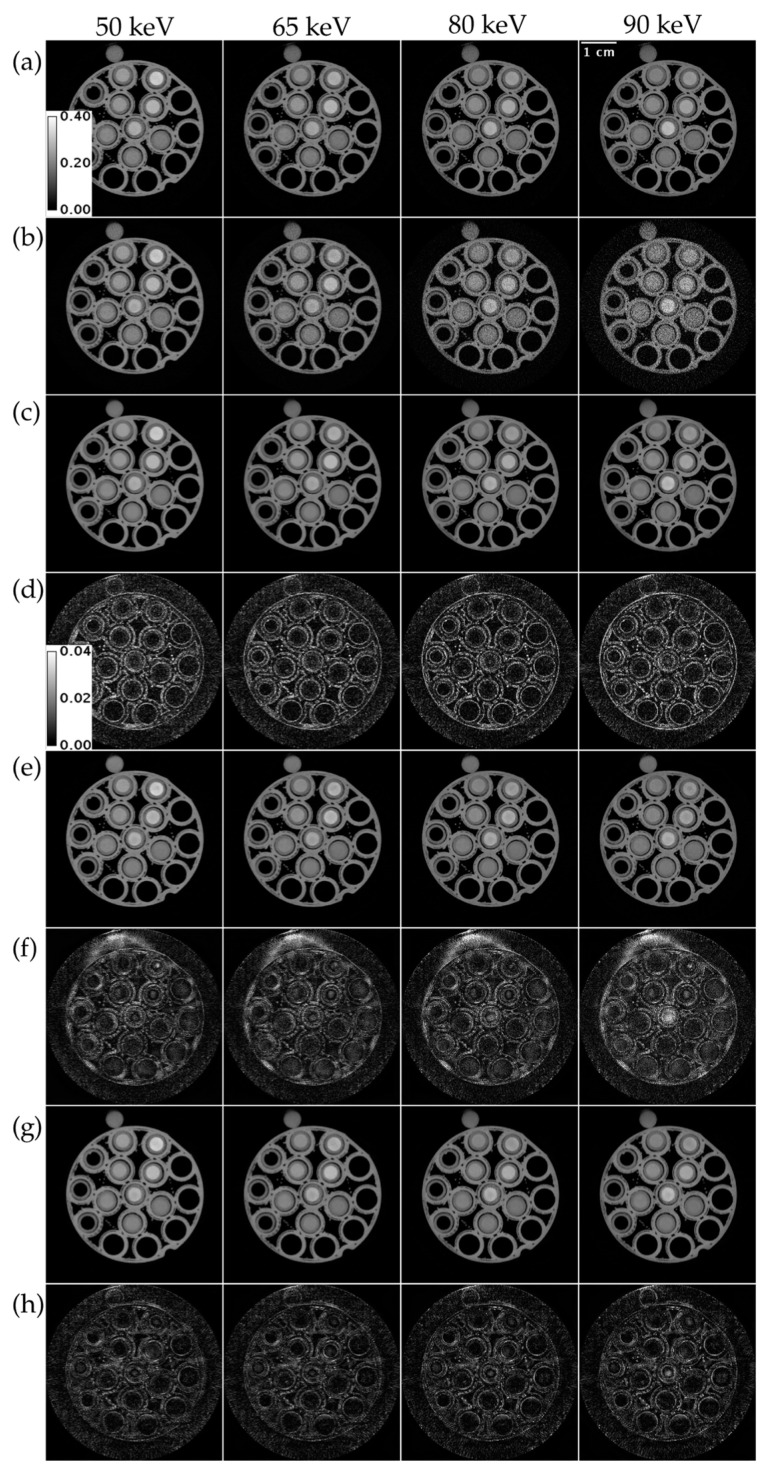
Reconstructions of a vials phantom scan with threshold settings at the Gd, Ta, and Bi K-edges. (**a**) Iterative RSKR reconstruction; (**b**) wFBP reconstruction; (**c**) ME NLM denoised reconstruction; (**d**) Absolute difference between (**c**,**a**); (**e**) UnetwFBP prediction; (**f**) Absolute difference between (**e**,**a**); (**g**) UnetU with custom loss prediction; (**h**) Absolute difference between (**g**,**a**). Calibration bars show the display window range of reconstructions and absolute difference maps in units of cm^−1^.

Figure 5 compares ME NLM, UnetwFBP, and UnetU with custom loss to iterative RSKR reconstruction in a test set scan of a vials phantom with threshold settings for the K-edges of Gd, Ta, and Bi. ME NLM (RMSE = 80.271 HU) denoises with low bias but causes blurring at edges, as shown in Figure 5d. Both U-net predictions show less edge blurring. However, UnetU with custom loss (RMSE = 50.605 HU) introduces much less bias in the vials than UnetwFBP (RMSE = 91.178 HU), as shown in Figure 5f,h.

**Figure 6:**
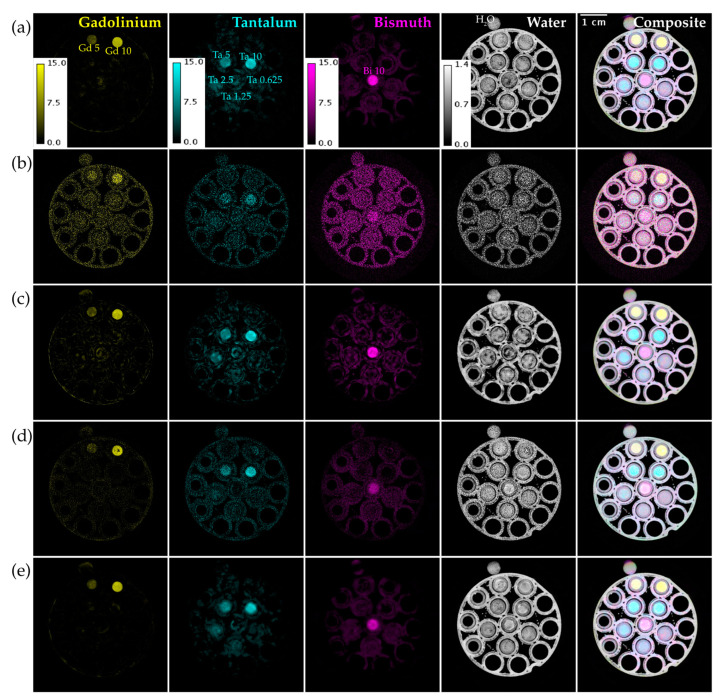
Material decomposition of a vials phantom scan with threshold settings at the Gd, Ta, and Bi K-edges. (**a**) From iterative RSKR reconstruction; (**b**) From wFBP reconstruction; (**c**) From ME NLM denoised reconstruction; (**d**) From UnetwFBP prediction; (**e**) From UnetU with custom loss prediction. Labels on the material maps in (**a**) indicate the locations of the water vial and vials with known concentrations (in mg/mL) of Gd, Ta, and Bi. Calibration bars show the display window range in units of g/mL for water and mg/mL for all other materials.

Figure 6 shows material decompositions of the reconstructions from Figure 5. The basis materials are the three contrast agents in the phantom (Gd, Ta, Bi) and water. We show both individual material maps in different colors and their composite. The decomposition from UnetwFBP prediction still has a lot of background noise, while both ME NLM and UnetU images result in low noise decompositions. However, the decomposition from ME NLM shows more artifacts and erroneous enhancement of the background due to excessive blurring of the reconstruction.

### 3.5. In Vivo Mouse Scan

**Figure 7:**
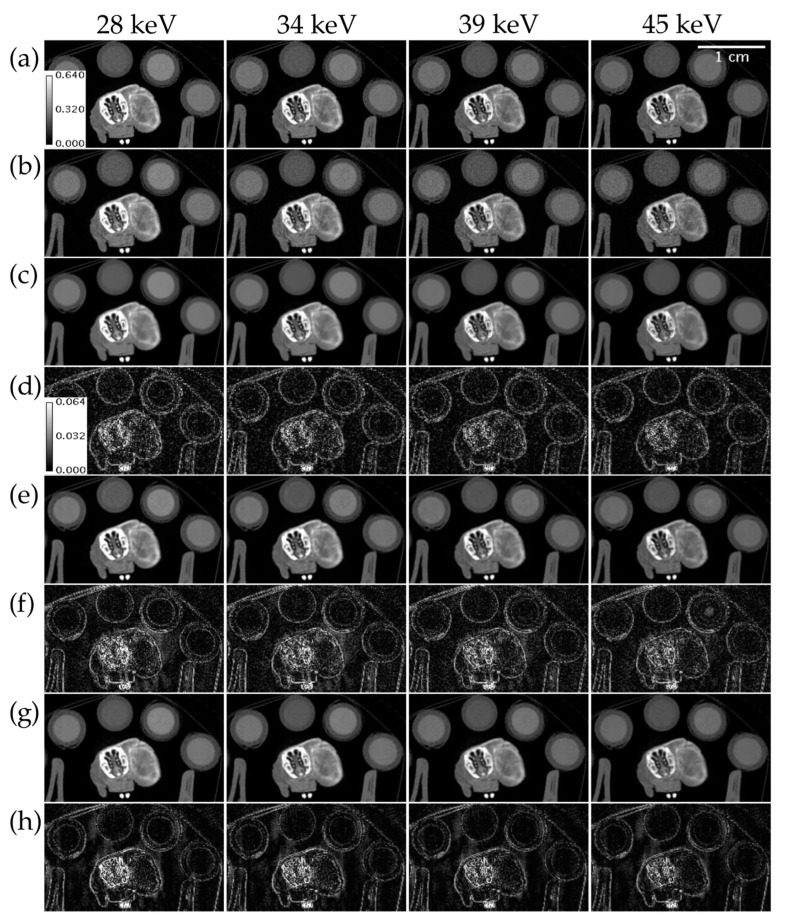
Reconstructions of an in vivo mouse scan with threshold settings at the I and Ba K-edges. (**a**) Iterative RSKR reconstruction; (**b**) wFBP reconstruction; (**c**) ME NLM denoised reconstruction; (**d**) Absolute difference between (**c**,**a**); (**e**) UnetwFBP prediction; (**f**) Absolute difference between (**e**,**a**); (**g**) UnetU with custom loss prediction; (**h**) Absolute difference between (**g**,**a**). Calibration bars show the display window range of reconstructions and absolute difference maps in units of cm^−1^.

Figure 7 compares ME NLM, UnetwFBP, and UnetU with custom loss to iterative RSKR reconstruction in a test set scan of an in vivo mouse with a tumor injected with I and Ba that was acquired with threshold settings not included in the training set. While ME NLM and both U-nets show some bias in high intensity regions such as the bone, UnetU with custom loss (RMSE = 41.524 HU) has lower error than both ME NLM (RMSE = 47.948 HU) and UnetwFBP (RMSE = 46.199 HU), due to superior noise reduction and preservation of edges.

**Figure 8:**
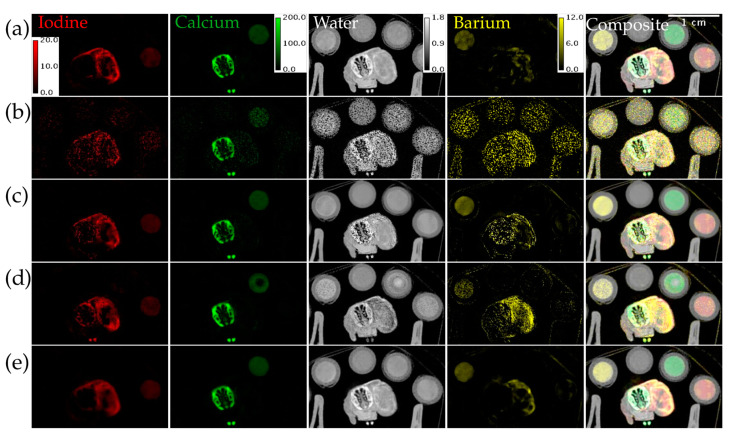
Material decomposition of an in vivo mouse scan with threshold settings at the I and Ba K-edges. (**a**) From iterative RSKR reconstruction; (**b**) From wFBP reconstruction; (**c**) From ME NLM denoised reconstruction; (**d**) From UnetwFBP prediction; (**e**) From UnetU with custom loss prediction. Calibration bars show the display window range in units of g/mL for water and mg/mL for all other materials.

Figure 8 shows material decompositions of the reconstructions from Figure 7. The decomposition from ME NLM has some noisy regions in the body of the mouse. The decomposition from UnetwFBP shows noticeable bias in the bone, tumor, and Calcium vial in all the material maps. The decomposition from UnetU appears to show some blurring, but comes closest to reproducing the decomposition from iterative RSKR reconstruction.

### 3.6. Performance at Different Dose Levels

**Figure 9:**
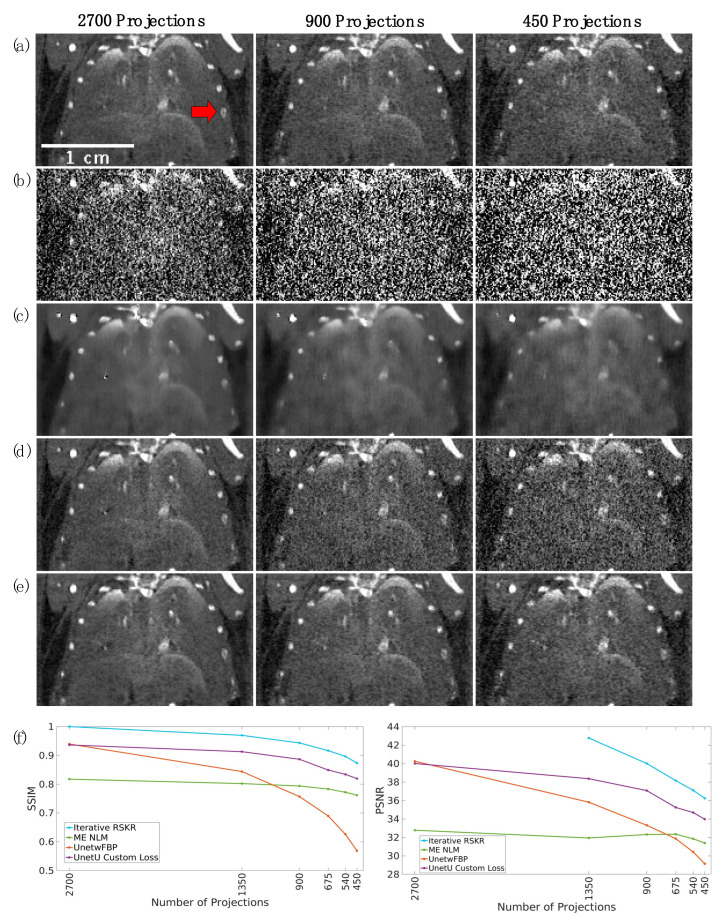
Performance of each denoising method at different dose levels in the highest energy threshold (60 keV) of an ex vivo mouse scan. (**a**) Iterative RSKR reconstructions; (**b**) wFBP reconstructions; (**c**) ME NLM denoised reconstructions; (**d**) UnetwFBP predictions; (**e**) UnetU with custom loss predictions; (**f**) SSIM (left) and PSNR (right) values for each denoising method at each dose level relative to the iterative reconstruction with 2700 projections. Since SSIM and PSNR values for iterative RSKR reconstruction at 2700 projections compare the reference image to itself, they have values of 1 and +∞, respectively. The calibration bar shows the display window range for reconstructions in units of cm^−1^. The red arrow in (**a**) indicates a rib bone that is blurred in ME NLM images and corrupted by noise in UnetwFBP predictions but preserved in iterative RSKR reconstructions and UnetU predictions.

Figure 9 displays coronal slices from the chest of an ex vivo mouse scan, reconstructed using varying numbers of projections (specifically, 2700, 900, and 450), thereby altering radiation dose. These images serve to exemplify the performance of wFBP, iterative RSKR reconstruction, ME NLM, UnetwFBP, and UnetU with custom loss at the highest energy threshold of 60 keV. ME NLM denoising significantly blurs the image, particularly at lower dose levels. UnetwFBP does not blur as much as ME NLM, but it is unable to provide sufficient noise reduction at lower dose. UnetU with custom loss and iterative RSKR reconstruction provide a much more optimal balance between noise reduction and spatial resolution. This qualitative observation is supported by quantitative results in part (f) of Figure 9, which shows that iterative reconstruction gives the highest SSIM and PSNR at all dose levels, while UnetU with custom loss gives the 2nd highest values for all dose levels except the full set of 2700 projections. It is worth noting that UnetU predictions have high SSIM and PSNR values in a coronal slice even though the network denoises axial slices.

## 4. Discussion

This work presents UnetU as a fast and adaptable denoising method for PCCT. Remarkably, UnetU can denoise a standard four-energy PCCT reconstruction 15 times faster than iterative reconstruction with RSKR. Results from the test set showed that UnetU with custom loss function outputs denoised images with higher spatial resolution than ME NLM and lower bias than UnetwFBP. In addition, UnetU with custom loss consistently has greater accuracy in the material decomposition than both ME NLM and UnetwFBP. Assessment of denoising performance at different dose levels (see Figure 9) showed that UnetwFBP has inadequate noise reduction and ME NLM severely degrades spatial resolution at low dose, while UnetU with custom loss can provide noise reduction at a wide range of dose levels with much less blurring than ME NLM. These results demonstrate that UnetU is a generalizable DL method that can quickly predict a good approximation of iterative RSKR reconstruction for a variety of acquisition settings with the same PCD.

There are a few limitations that merit further discussion. Like all PCDs, the detector used to acquire our training and testing sets is affected by spectral distortions such as charge sharing and pulse pileup [2]. The iterative RSKR reconstruction algorithm used to generate our training labels does not correct for these effects. Future work could further improve the accuracy of our denoising approach by using PCCT simulations, a model-based correction, or EID scans to make iterative RSKR reconstructions without spectral distortions as training labels. In recent work, we have improved PCCT material decomposition accuracy by training a U-net with material maps derived from iterative reconstruction of high-dose, multi-energy scans with an EID [30]. In a similar fashion, we can create training labels for PCCT denoising by converting multi-energy EID material decompositions into PCD energy threshold images by using material basis functions. Although our UnetU method results in greater quantitative accuracy than ME NLM and UnetwFBP in both the reconstruction and material decomposition domains, we acknowledge that UnetU predictions still have some bias in high-intensity regions of the reconstructions. Additional refinements to the input preprocessing approach, loss function, or CNN architecture could help address this issue.

In addition to these specific limitations, there are more general challenges associated with a DL approach that should be considered. The availability of training data poses a significant challenge, as DL-based denoising methods typically require high-quality data, which in CT often necessitates higher-dose acquisitions. However, our approach leverages iterative RSKR reconstructions as training labels, eliminating the need for additional high-dose images and simplifying the data acquisition process. DL models also demand substantial computational resources for training and inference, which can preclude their use in resource-limited settings. In our study, we were fortunate to have access to high-performance, multi-GPU computers to facilitate our DL training and evaluation. DL models’ inherent complexity can impede interpretability, making it challenging to understand the decision-making process. However, the use of a U-net CNN architecture in our approach mitigates this challenge to some extent, since it has far fewer weights than other more complicated networks. This allows insights into the denoising process and contributes to transparency. Finally, the robustness and adaptability of DL models to different data acquisition protocols needs careful investigation. By training and testing our U-net models on diverse datasets encompassing several PCCT energy threshold settings, we aimed to assess their ability to handle variations encountered in practical scenarios. However, a large test set with systematic variation in multiple acquisition parameters would allow a more complete understanding of the extent to which DL approaches such as UnetU are generalizable.

Nevertheless, our work establishes an effective supervised DL approach that enables fast approximation of iterative RSKR reconstruction and accurate material decomposition for a wide variety of PCD scan protocols. A generalizable denoising approach such as UnetU assists in imaging studies involving novel contrast agents, opening new avenues for research in spectral CT.

## 5. Conclusions

In this work, we presented UnetU, a deep learning approach for fast PCCT denoising that combines a standard U-net CNN architecture with novel input preprocessing and a complex loss function to provide accurate denoising and material decomposition at a variety of PCD energy threshold settings. We determined that UnetU denoises a typical wFBP reconstruction 15 times faster than iterative RSKR reconstruction. UnetU gave lower average test set RMSE (relative to iterative RSKR reconstruction) than UnetwFBP and ME NLM in reconstructions and material maps. This quantitative result was supported by qualitative results, indicating that UnetU provides the best approximation of iterative RSKR reconstruction in both the reconstruction and material decomposition domains.

## Figures and Tables

**Table 1:** Summary of scan protocols for training and testing sets. Samples scanned for this study included in vivo mice, ex vivo mice, and phantoms containing vials of known material concentration.

PCD Thresholds (keV)	Training Sets	Validation Sets	Test Sets	Contrast Materials	kVp	mA	Dose (mGy)	Types of Samples Scanned
25, 34, 50, 60	3	0	1	I, Gd	80	4	36	in vivo, ex vivo
25, 34, 50, 80	2	1	1	I, Au	125	2.5	38	ex vivo
25, 28, 34, 40	2	0	1	I	80	4	36	ex vivo, phantom
50, 65, 80, 90	2	0	1	Gd, Ta, Bi	125	2.5	38	ex vivo, phantom
28, 34, 39, 45	0	0	1	I, Ba	80	4	36	in vivo

## Data Availability

The data and code will be shared on https://gitlab.oit.duke.edu/rohan.nadkarni/unetu_pcctdenoising/ (accessed on 1 July 2023).
